# Qingfei Xiaoyan Wan alleviates asthma through multi-target network regulation

**DOI:** 10.1186/1472-6882-13-206

**Published:** 2013-08-06

**Authors:** Zhenying Zhao, Yingbo Miao, Pengwei Pan, Binfeng Cheng, Gang Bai, Hong Wu

**Affiliations:** 1 College of Pharmacy, Nankai University, Tianjin, People's Republic of China; 2 Department of Pharmacy, Tianjin People’s Hospital, Tianjin, People's Republic of China; 3 Department of Pharmacology, Tianjin Medical University, Tianjin, People's Republic of China

**Keywords:** Asthma, Traditional Chinese Medicine, Microarray, 2D- electrophoresis, MS identification, Multi-target network

## Abstract

**Background:**

Qingfei Xiaoyan Wan (QFXY), a traditional Chinese formula, is widely used for relieving cough, asthma, upper respiratory tract infection, bronchitis, pneumonia, and etc. in clinic. Comparing with other anti-asthma drugs, it is characterised with moderate and persistent efficacy as well as few side effects, however, the underlying action mechanism still remains elusive. This study aimed to identify QFXY multi-target network regulation as an asthma controller.

**Methods:**

This study established asthma model induced by histamine phosphate and acetylcholine chloride (His&Ach) in guinea pigs, which then were administered orally with QFXY. Hematoxylin-Eosin staining sections were applied for evaluating QFXY effect. In both Model and QFXY groups, customized microarrays and 2D electrophoresis were adopted to detect differentially expressed genes (diff genes) and proteins (diff proteins) respectively, and some diff proteins were identified with MALDI-TOF/MS. The checked diff genes and proteins underwent Cluster, GO and KEGG analysis. Based on GAD and HPRD databases, QFXY-asthma target regulation network was constructed.

**Results:**

His&Ach-induced asthma model of guinea pigs was established. HE sections presented anti-inflammation and anti-remodelling effects of QFXY. Comparing with the Model group, 55 diff genes and 6 diff proteins were identified in QFXY group. Validation by qPCR and Western blot showed the microarray and 2D data reliable. Furthermore, QFXY-asthma target regulation network was achieved.

**Conclusions:**

A primarily combined genomic and proteomic screening of QFXY targets displayed a series of candidate genes and proteins, which indicated that the effect of QFXY relied on the combined mechanism, anti-inflammation and anti-remodelling, as well as influencing signal transduction in vivo.

## Background

Asthma, as defined in 2008 by the Global Initiative for Asthma (GINA), is an inflammatory disorder of the airways in which many cells and cellular elements play roles [1]. Bronchial hyperactivity associates with inflammation, that together with an external or environmental insult, on a vulnerable bronchial epithelial structures, generates tissue remodelling and respiratory functional impairment [2]. Asthma is not a curable disease at the present time [3,4]. However, with proper treatments, the risk of mortality for asthmatic individuals could be comparable to that of the general population. Presently, the treatment of asthma includes a dual focus: the short-term treatment of acute symptoms with bronchodilators, and together with the prevention or eventual reversal of chronic inflammation using anti-inflammatory drugs [5].

Medications to treat asthma can be classified as controllers or relievers. Controllers are medications taken daily on a longterm basis to keep asthma under clinical control chiefly through their anti-inflammatory effects. Relievers are medications used on an as-needed basis, which act quickly to reverse bronchoconstriction and relieve its symptoms [1,6]. The major medications in asthma management include bronchodilator β_2_-agonists,anti-inflammation inhaled corticosteroids, leukotriene modifiers and theophyllines. The use of rapid-acting β_2_-agonists in long period may lead to relative refractoriness to β_2_-agonists [7]. Long-acting inhaled β_2_-agonists, including formoterol and salmeterol, should never be used as monotherapy for asthma as these medications do not appear to influence the airway inflammation in asthma. They are most effective when combined with inhaled glucocorticosteroids, and this combination therapy is the preferred treatment when a medium dose of inhaled glucocorticosteroid alone fails to achieve control of asthma [8,9]. Inhaled glucocorticosteroids are currently the most effective anti-inflammatory medications for the treatment of persistent asthma. The systemic side-effects of long-term treatment with high doses of inhaled glucocorticosteroids include easy bruising, adrenal suppression and decreased bone mineral density and etc. When the drugs are discontinued, deterioration comes out within weeks to months in proportion of cases [1]. Leukotriene modifiers are associated with dose reductions of inhaled glucocorticosteroids, while monitoring of liver tests is recommended during their treatment for the underlying liver toxicity [10]. Theophylline, a bronchodilator, when given in a lower dose, has modest anti-inflammatory properties, but needs proper monitoring for its narrow therapeutic range [11]. As mentioned above, there is a consistent need to explore novel effective anti-inflammation and bronchodilator drugs, especially suitable for the senior and children or chronic patients.

QFXY is originated from a famous Traditional Chinese Medicine (TCM) formula “Maxing Shigan Decoction”. It has been experimentally improved, consisting of eight materia medicas, Ephedra Herba, Saigae Tataricae Cornu, Pheretima, Arctii.

Fructus, Lepidii Semen, Bovis Calculus Artifactus, Armeniacae Semen Amarum and Gypsum Fibrosum. Since decades of extensive clinical practice, QFXY has shown significantly therapeutic effects on dissolving phlegm as well as relieving cough, asthma, upper respiratory tract infection, bronchitis, pneumonia, and etc., but its underlying action mechanism still remains elusive [12].

Our previous study revealed QFXY composition with UPLC/Q-TOF-MS, consisting of 55 ingredients including 27 absorbable constituents [13]. In this study His&Ach-induced asthma model in guinea pigs was established, and QFXY was administered orally. HE stained sections were applied for QFXY effect evaluation. Customized microarrays and 2D electrophoresis (2DE) were adopted to detect differentially expressed genes and proteins (“diff genes” or “diff proteins” for short) respectively. Some diff proteins were identified with MALDI-TOF/MS. Cluster, GO and KEGG analyses enrich the functions and pathways of the diff genes and proteins. Based on asthma related genes from GAD and HPRD databases, the interaction network of all diff genes (including both diff genes from microarrays and diff genes blasted from diff proteins from 2DE) with asthma related genes was achieved, which indicated QFXY had multi-target regulation on asthma. Some detailed ingredients of QFXY may become candidate anti-asthma drugs in the future.

## Methods

### Drugs and animals

QFXY pills (Lot. 100512) were provided by Tianjin Zhongxin Pharmaceutical Group. Guinea pigs of England specie, (300 ± 20) g, male and female, were purchased from Beijing Vital River Laboratory Animal Technology Co., Ltd. The animals were housed at 22 ± 2°C with 55 ± 10% humidity, 12 h light/dark cycle, and had free access to species-specific food and tap water. All experiments were carried out according to the Guide for the Care and Use of Experimental Animals. Studies were approved by the Institute Committee of the Animal Care of Nankai University, China.

### Protocol of asthma model

Inside a container, guinea pigs were given the mixed solution of 0.1% histamine phosphate and 2% acetylcholine chloride for 10 s with ultrasonic sprayer (0.5 mL/min). The time when asthma occurred was recorded [14]. The asthmatic guinea pigs were randomized into 3 groups, QFXY2 (397.5 mg/100g BW), QFXY1 (or “Q” for short) (132.5 mg/100g BW) and Model group (“M” for short) (n=6 for each group), were administrated orally with QFXY and normal saline respectively for 7 days. Again, guinea pigs were put into the glass cup and given 0.1% histamine phosphate for 10s, and prolonged period of asthma was recorded. There was another group without any treatment as the Normal group (“N” for short) for the following pathological sections and microarrays. The lung tissues of guinea pigs prepared for further experiments.

### Pathological analysis

HE sections of bronchial and lung tissue of guinea pig were conducted according to the regular methods [15]. Briefly, the fresh lung tissue samples were fixed in 10% formalin, and embedded in paraffin. Samples were cross-cut into 40-50 slices and the thickness of 4-5μm. The slices were stained by Hematoxylin-Eosin (HE). Finally, the stained sections were observed in light microscope (100× and 40×).

### Microarray procedures and data analysis

Total RNA of 50mg lung tissues of each group was extracted [16] with Trizol (Invitrogen, USA), chloroform, isopropanol, 75% ethanol, and purified using Nucleo Spin RNA Clean-up Kit (MN, Germany). RNA concentration and integrity were determined by UV-1800 Spectrophotometer (Shimadzu, Japan) and agarose gel electrophoresis. Four Guinea pigs gene expression chips were customized (Agilent Technologies, USA). The dual-channel chips were scanned with LuxScan 10KA dual channel laser scanner (CapitalBio, China). In the primary hybridization profiles, cy5 in red, cy3 in green, three chips were QFXY/Normal, one chip was Model/Normal. The relative differential ratio of signal strength, (QFXY/Normal)/(Model/Normal) presented the relatively varied gene expression of QFXY/Model. The relative ratios were for the following SAM (Significance Analysis of Microarrays) analysis for diff gene screening. 2_3, 2_4 and 2_9 were of the QFXY group. RNA of the QFXY group was isolated from each sample individually and was not pooled. But RNA samples from the Model group and Normal group were pooled to reduce biological differences [17]. SAM One Class method was adopted for the analysis of diff genes. Standard criteria for diff genes were |Score (d)| ≥2 and Fold Change≥2 (or ≤0.5). Cluster 3.0 was used with the hierarchical average linkage algorithm to get a heat map. In PubMed, the reference sequences of guinea pig were blasted to human genes, with the E value less than 1e-5, and the similarity between two sequences spanned over half sequence length [18]. The human genes were imported Molecule Annotation System (MAS3.0) [19] for GO and Pathway analysis.

### 2D-electrophoresis and MS identification

Proteins were isolated from 20 mg lung tissues of each group with RIPA (Radio-Immunoprecipitation Assay) Lysis Buffer (SolarBio, China) containing 1Mm PMSF (Phenylmethanesulfonyl fluoride) for 15min lysis on ice and centrifuged in 10000g. Protein concentration was diluted to 2mg/ml by Bradford method [20]. In 2D-electrophoresis instrument (BIO-RAD), pH 3-l0 precast IEF strips, 0.7 mg sample loading, total v • h 80000, 120 g/L gel for SDS-PAGE, and Coomassie brilliant blue staining method was adopted. The GS-800 scanner was used for acquiring image, with PDQuest 7.1 software for dot cutting, editing, detecting and matching. MS analysis providing purity, molecular weight, amino acid sequence, composition of peptide fragments, as well as the database support, differential proteins can be identified. Based on the MS report, protein score greater than 60 or single peptide score over 30 is more reliable. If more than one protein scored over 60, the top ranked is more credible. C.I. % over 95% is also reliable criterion. Besides, we also compared the theoretical protein molecular weight and isoelectric point with those we obtained in 2DE analysis. Furthermore, the diff proteins can be blasted into genes for further study.

### Quantitative real-time PCR and data analysis

Validation of changes of diff genes in guinea pig lung tissues was carried out by real-time quantitative polymerase chain reaction (qPCR). First, total RNA was converted to cDNA using High Capacity cDNA Reverse Transcription Kits (ABI, USA). According to the reference sequences of guinea pig, the primers used are as follows: RHO (forward: 5’-CACCGCTCAACTACATCCTGC-3’, Reverse: 5’- GCCCGAAGACGAAGTATCCA-3’), GNB1 (forward: 5’- TCCATCTACAATCTGAAGACTCGC -3’, Reverse: 5’- TAGTCTGGTGTCGGGAGCG -3’), MAPK3 (forward: 5’- CTCAACCACATTCTGGGTATCC -3’, Reverse: 5’- CCACCTTAGTCTTAGAGGGCAGA -3’), CLU (forward: 5’- GGATGAAGGACCAGTGCGAG -3’, Reverse: 5’- CGTGGAGACATGGAGATAGGC -3’), ENO1 (forward: 5’- CAGAAGTCACAGCCAGCGTG -3’, Reverse: 5’- CTTTGAGCAGCAGGCAGTTG -3’), HSP90α (forward: 5’- GGCAGAGGCTGATAAGAACG -3’, Reverse: 5’- AGTCATCCCTCAGCCAGAGA -3’), SERPINE2 (forward: 5’- CAGGGTCAGAAAACCTCCAT -3’, Reverse: 5’- CTGCCCCATGAATAACACAG -3’), GAPDH (forward: 5’- GAACATCATCCCCGCATCC -3’, Reverse: 5’- GTCCTCGGTGTAGCCCAAGA -3’). Real-time PCR for quantitative assessment of mRNA expression was performed on LightCycler 2.0 (Roche, Swiss) with GoTaq qPCR Master Mix (Promega, USA) according to the manufacturer’s protocol. The PCR conditions were as follows: 94°C for 2 min, followed by 40 cycles of amplification (95°C for 15 s, 55°C for 30 s, 72°C for 30 s), and a dissociation stage. 2^-ΔΔCt^ method was applied for data analysis.

### Western blot of Hsp90

The protein sample (40 μg) was separated by 12% denaturing SDS-PAGE and blotted onto a nitrocellulose membrane. After electrophoresis, the proteins were transferred to nitrocellulose membrane by electrophoretic transfer system. The membranes were blocked in 5% skimmed milk in TBS for 1h, and then incubated with primary antibody (anti-Hsp90 at 1:2000 dilution and anti-β-actin antibodies at 1:10000 dilution) (Wuhan Boster, China) overnight at 4°C. The membranes were incubated for 2 h in horseradish peroxidase-conjugated goat anti-rabbit secondary antibody (1:2500 dilution) for 2 h. Antigen-antibody complex was visualized by enhanced chemiluminescence reagents Supersignal (Pierce, Rockford, IL). For quantification, Quantity One software (Bio Rad, USA) was used.

### QFXY-asthma target network construction

Human protein interaction data were sourced from Human Protein Reference Database (HPRD) [21] as the background. Asthma related genes from Genetic Association Database (GAD) [22] were annotated to the background network. Those nodes having direct interactions with asthma genes were used to build an asthma disease subnetwork. Keep the possibly same interactions in the subnetwork and HPRD network overlapped. By Cytoscape (Ver. 2.6.0) [23], the diff genes (from both microarray and 2DE data) were annotated in the asthma disease subnetwork and achieved QFXY-asthma target network.

### Statistics

Data were presented as Mean±SD. The significance in mean values was analyzed by *t* test for 2 groups and by analysis of variance (ANOVA) with least squares difference post-hoc test for more than 2 groups. Values were considered statistically different at *p*<0.05.

## Results

### Histopathological results

**Figure 1:**
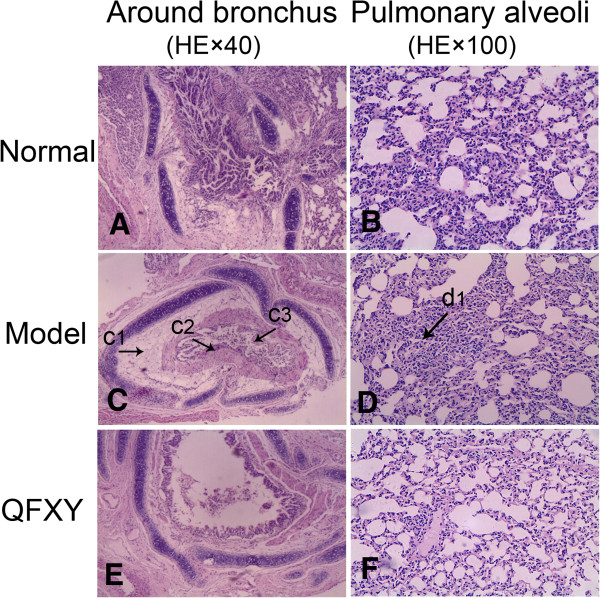
**The pathological sections of each group (HE×40 and HE×100). (A**&**B)** Normal group, **(C**&**D)** Model group, **(E**&**F)** QFXY group. **A**, **C** and **E** showed lung tissues of guinea pigs around bronchus; **B**, **D** and **F** displayed around pulmonary alveoli. Comparing with the Normal group and QFXY group, obvious inflammatory cell infiltration in mucosa and submucosa seen in Model group. Arrow c1 displayed submucosa of spongiform vacuoles degeneration, necrosis; Arrow c2 displayed edema of tracheal mucosa, thickened bronchial walls, more goblet cells; Arrow c3 displayed narrowed or even blocked bronchial lumen, mucous plugs, necrosis mucosa; Arrow d1 displayed alveolar tissue consolidation.

To test the QFXY effect, the pathological sections of lung tissues were stained by HE demonstrated in Figure 1. In the Model group (Figure 1C and Figure 1D), pathological sections showed significant edema of tracheal mucosa, presenting mucosa epithelial cells swelling, some epithelial cells in spongiform vacuoles degeneration, necrosis and loss, and more goblet cells. Narrowed or even blocked bronchial lumen, thickened smooth muscles of the bronchial walls, and mucous plugs were visible and bronchial vascular congestion and angiogenesis, and inflammatory cell infiltration in mucosa and submucosa as well as perivascular tissues. In the Normal group (Figure 1A and Figure 1B), neither was obvious edema in airway mucosa, nor inflammatory cell infiltration in airway and vascular vessels. Bronchial tube cavity is smooth and unblocked. Comparing with the Model group, the QFXY group (Figure 1E and Figure 1F) has obvious change in bronchial lung structure, more similar to the Normal group, which preliminarily showed sound effect.

### Microarray analysis and qPCR validation

**Figure 2:**
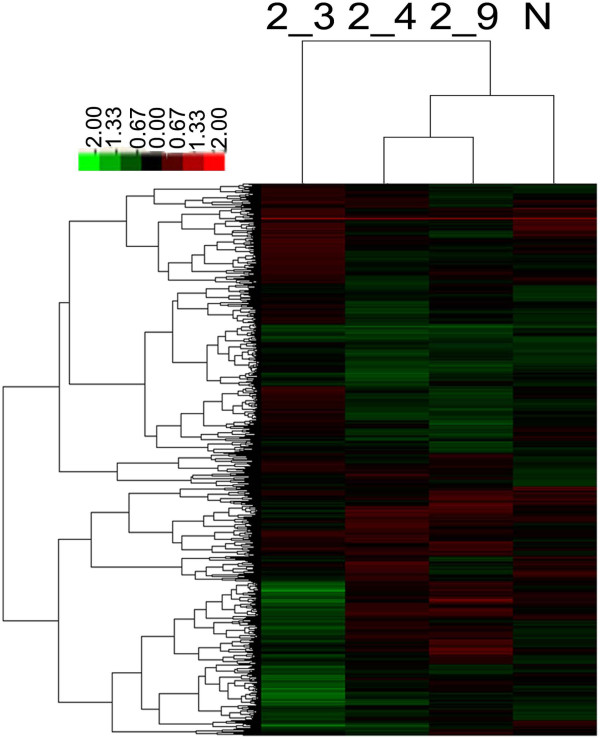
**Heat map of the microarrays by hierarchical clustering.** Heat map is a visual diagram, in which the vertical ordinates showed various chips, and the horizontal ordinates showed genes expressed in all chips. 2_3, 2_4, 2_9 were of the QFXY group. N was the Normal group. Red indicated a minimum two fold increase in expression; green represented a minimum two fold decrease in expression, compared between the Normal control and QFXY treatments.

**Figure 3:**
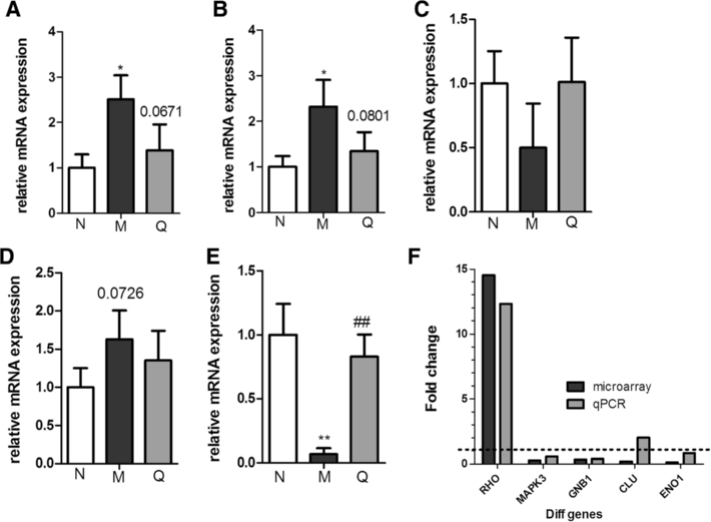
**qPCR confirmation of the diff gene expressions with GAPDH as internal reference.** Results were expressed as Mean±SD, ^*^p < 0.05, ^**^p < 0.01 vs. Control group; ^#^*p* < 0.05, ^##^*p* < 0.01 vs. Model group.

In our study, guinea pig cDNA microarrays were customized using the sequences as many as we could archive in NCBI EST database [24] (19911EST and 930CDS), which assemble can be used as a microarray design template for guinea pig. SAM analysis screened 55 diff genes of guinea pig, with 14 up-regulated and 41 down-regulated, see Additional file 1. Hierarchical Cluster analysis generated a heat map, shown in Figure 2, generally revealing gene expression module comparison of the samples. As shown in the Heat Map of the Figure 2, 2_4 and 2_9, the expression profile of the QFXY group had more similarity to that of the Normal group, which suggested that with the QFXY treatment, the overall gene expression profiles were inclined to the normal level, indicating the mitigation and improvement of asthma. The gene expression was verified with qPCR, seen in Figure 3A- 3E. The correlation of expression level in microarray and qPCR seen in Figure 3F.

### 2DE, MS identification and validation

**Figure 4:**
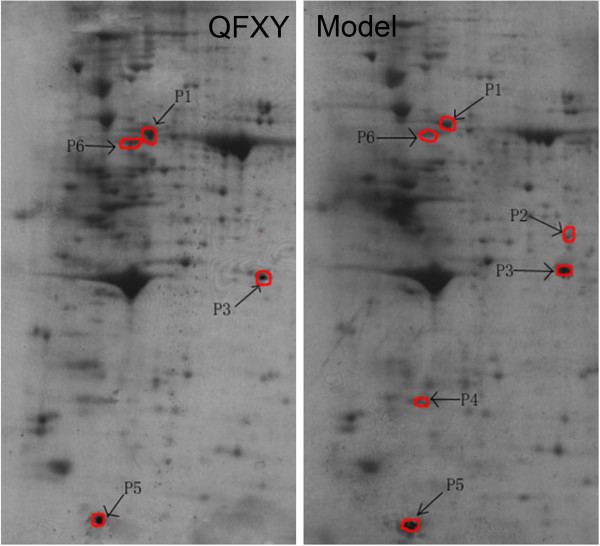
**The 2DE results of QFXY and Model group.** Identified diff proteins were marked by arrows in 2DE of QFXY group and Model group.

**Figure 5:**
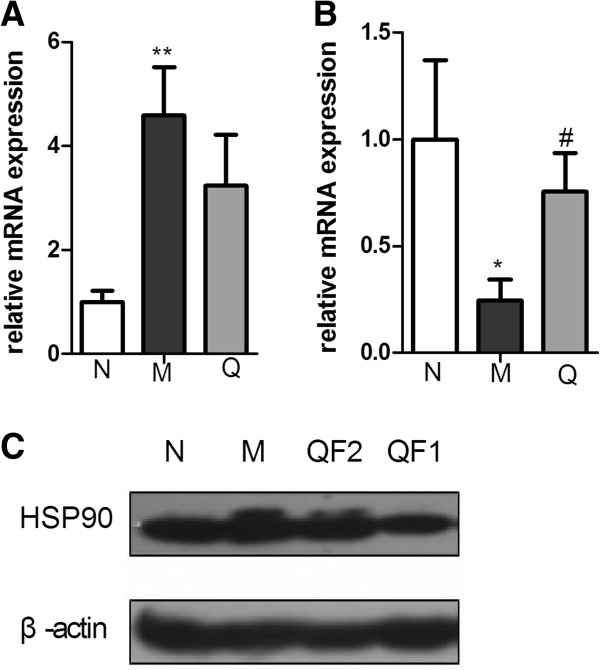
**qPCR and Western blot conformation of Hsp90 and Serpin with QFXY treatment.** qPCR validation with GAPDH as internal reference **(A, B)**. Western blot validation with β-actin as internal reference **(C)**. Results are expressed as the Mean±SD, *p < 0.05, **p < 0.01 vs. Control group; ^#^*p* < 0.05, ^##^*p* < 0.01 vs. Model group. (QF2-397.5mg/100g BW, QF1-132.5mg/100g BW).

2DE results were seen in Figure 4. Some diff proteins were identified using MALDI-TOF/MS seen in Table 1. Due to limited research data of guinea pig, diff proteins were blasted into human proteins as well as relevant genes. Protein expression was validated with qPCR and Western blot displayed in Figure 5. The expression level of Hsp90 decreased and Serpin increased with QFXY treatment comparing with the Model group.

**Table 1:** MS identification of some diff proteins

**Diff proteins**	**Protein score**	**Protein score C**. **I**. **%**	**Average 2DE data**		**Diff ratio (QFXY/Model)**	**Human homolog**
			**QFXY**	**Model**		
P1	946	100	55703.9	10498.7	5.31	heat shock protein Hsp90-alpha isoform 2
P2	164	100	(−)	2331.6		macrophage-capping protein
P3	151	100	15264.5	25045.8	0.61	microfibrillar-associated protein 4
P4	278	100	(−)	3976.3		gamma-actin
P5	348	100	40773.4	20867.1	1.95	lamin-B1
P6	347	100	16005.9	2196.3	7.29	serine proteinase inhibitor A3K

### GO and pathway enrichments

There are few guinea pig research data of definite functions of genes and signal pathways. In NCBI, we blasted 55 diff genes of guinea pig and got 27 human homologues, see Additional file 2. The molecular function, biological process and cellular component of the 27 diff genes see Additional file 3, especially involved in such biological processes as signal transduction, protein phosphorylation, stress response and etc. The diff genes participate in some pathways, see Additional file 4. Sourced from KEGG [25], GenMapp [26] and BioCarta [27], diff genes participated in several common signal pathways, some of which were involved in inflammation (Hs_TNF-alpha-NF-κB_NetPath_9, Prostate cancer pathway), cell movement and proliferation as well as airway remodelling of the cytoskeleton and extracellular matrix (Hs_Regulation_of_Actin_Cytoskeleton, extracellular matrix, intermediate filament cytoskeleton, structural constituent of cytoskeleton, actin binding, actin cytoskeleton), multi-level signaling protein folding (protein folding, unfolded protein binding), cell adhesion and signal transduction (Adherens junction, Focal adhesion), and so on. Important genes involved include HSP90A1, SERPINA1, MAPK3, ACTG1, VIM, TNNT2, GNB1, CRYAA, CRYAB, COL4A2, COL1A2 and so on. The compiled file and detailed pathways see Additional file 5.

### QFXY-asthma target network

**Figure 6:**
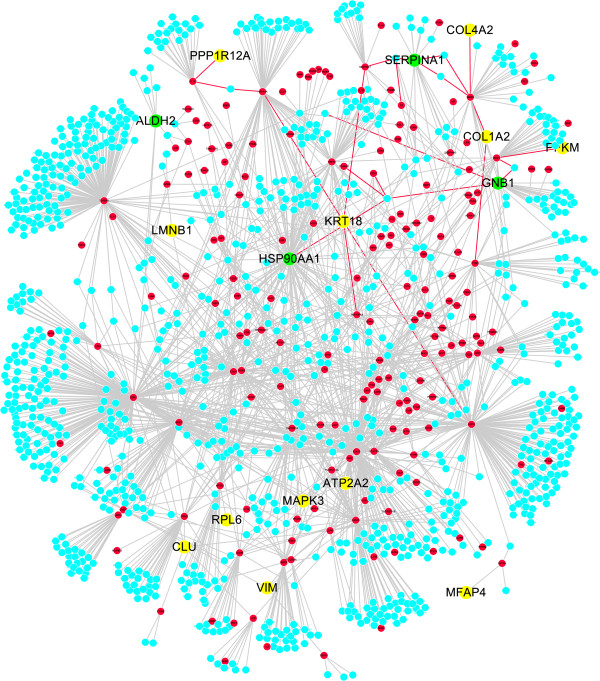
**QFXY**-**asthma target network based on interactions between all diff genes and asthma related genes.** Red for known asthma genes (resourced from HRPD&GAD), green for diff genes (including those blasted from diff proteins), yellow for diff genes which had direct interactions with asthma genes, blue for other genes directly interacting with asthma genes.

A network containing 1214 nods and 1886 interactions was constructed as QFXY-asthma target network. In Figure 6, red for known asthma genes (sourced from HRPD&GAD), green for diff genes (including those blasted from diff proteins), yellow for diff genes which had direct interactions with asthma genes, blue for other genes directly interacting with asthma genes. In total, the network contained 16 diff genes, 182 asthma genes (from databases), and 1016 genes directly interacting with asthma genes.

## Discussion

Studies in animal models form the basis for our current understanding of the pathophysiology of asthma, and are central to the preclinical development of drug therapies [28]. Guinea pigs have been the most commonly used small animal species in preclinical studies related to asthma and COPD [29]. β_2_-adenoceptor agonists and antimuscarinic drugs prevent antigen-induced bronchoconstriction in actively sensitized guinea pigs in a dose-dependent manner. Histamine is the major mediator in guinea pigs but not in humans [30]. Asthma is a complex disease defined by reversible airway narrowing, acute and chronic airway inflammation, airway hyperresponsiveness (AHR) and airway tissue remodelling, in which accumulation of airway smooth muscle (ASM) is a prominent and widely reported feature [31]. In the pharmacodynamics study, the prolonged asthma time [12] and HE sections showed that QFXY had significant effects on asthma, reducing edema in airway mucosa and inflammatory cell infiltration in airway and vascular vessels. They were also beneficial to reducing airway remodelling.

Among up-regulated genes, the fold change of RHO almost ranked top (Additional file 2). Among down-regulated genes, CLU and ENO1 had greater changes. Among 2D results, fold changes of Hsp90 and Serpin were of greater change (Table 1). Besides, references and literatures about every diff molecules were retrieved, of which some were related to the process of inflammation or asthma or lung diseases, such as GNB1 [32,33], MAPK3 [34-37]. Altogether, with the combined consideration of fold changes and references, these genes (RHO, CLU, ENO1, GNB1, MAPK3) and proteins (Hsp90, Serpin) were selected for validation test.

The GO annotation suggested that QFXY might influence the inflammation, signal transduction, stress response, the apoptosis of endothelial and bronchial cells. Pathway analysis revealed that different genes were involved in the signaling pathways, including focal adhesion pathway, cell-extracellular matrix interactions pathway, TGF-beta signaling pathways, NK cell-mediated cytotoxic pathway and so on, which are all related with cell signaling, inflammation, mast cells and NK cells. Many asthma drugs also participated in those pathways in variety of mechanisms, targeting kinases, receptors or related proteins, affecting inflammation response, mitosis, angiogenesis, apoptosis, and anti-oxidation, to play a role in asthma. The qPCR change profile was basically in line with the microarray results, proving the reliability of microarray data. The commonly shared signal pathways of diff genes and diff proteins combined the genomics and proteomics together, to manifest the underlying mechanism of QFXY effects.

The Mapk3/Erk signaling cascade is a central Mapk pathway that plays a role in the regulation of various cellular processes such as proliferation, differentiation, development, and inflammation reactions and etc. [34-37]. Inhibition of this kinase strongly decreased the expression of pro-inflammatory genes encoding growth-regulated proteins (Gro-a, Gro-b and Gro-g) and interleukins (IL-1β, IL-6 and IL-8) [36]. Mapk can participate in the regulation of NF-κB transcriptional activity [38,39]. Our previous study also presented decreasing erk expression and NF-κB inhibition [13].

Hsp90, as a molecular chaperone, has interactions with proteins, such as Akt and Raf-1 [40]. Akt is a downstream effecter molecule of phosphoinositide 3-kinase and is thought to mediate many immune and inflammatory responses. It is also involved in the activation of NF-κB [41]. Amino acid residues 229–309 of Akt were involved in the binding to Hsp90 and amino acid residues 327–340 of Hsp90 β were involved in the binding to Akt [42]. Hsp90 plays an important role in maintaining Akt kinase activity. In our study, 2D and Western blot showed decreased Hsp90 after QFXY treatment, as well as less NF-κB activity [13], indicating QFXY may affect the binding of Hsp90 and Akt, which needs further confirmation.

GTP-binding protein beta1 subunit gene (GNB1), its up-regulation appears to be one of the candidate processes of sensitization. It also has NF-κB recognition sites [32]. The Ectodysplasin is involved in binding to its ligand EDA-A1 and activates the NF-κB intracellular signaling pathway by interaction through its death domain with the adaptor protein EDARADD [33]. Down-regulated GNB1 and EDARADD gene expression decreased NF-κB activity for anti-inflammation.

Serpins form an enormous superfamily of 40-60-kDa proteins found in almost all types of organisms. Most have evolved to finely regulate complex proteolytic pathways, such as blood coagulation, fibrinolysis, and inflammation [43,44]. α1-antitrypsin (AAT) is an archetype member of the serpin supergene family. The reduced serum levels of AAT contribute to the development of chronic obstructive pulmonary disease (COPD). In addition to protease inhibition, AAT shows anti-inflammatory, immunomodulatory and antimicrobial properties [45,46]. SerpinA1 is an endogenous anti-inflammatory factor, and its anti-inflammatory effects may be mediated through antioxidant activity [47]. Compared with the Model group, the HE sections of the QFXY group showed less inflammation and mucosa hyperplasia, and the 2D and qPCR proved higher SerpinA1 expression, which indicating specific ingredients in QFXY can activate SerpinA1.

Asthma is a disease characterized by persistent inflammation and structural changes in the airways referred to as airway remodelling., including smooth muscle hypertrophy, goblet cell hyperplasia, subepithelial fibrosis, and angiogenesis. Vascular remodelling in asthmatic lungs results from increased angiogenesis, mediated by vascular endothelial growth factor (VEGF). In addition, VEGF induces allergic inflammation, enhances allergic sensitization, and has a role in Th2 type inflammatory responses [48]. Matrix GLA protein (MGP) has a role in endothelial cell (EC) function [49]. MGP modulates the activity of transforming growth factor (TGF)-β superfamily, which is critical for morphogenesis and development [50]. MGP can stimulate VEGF expression through increased TGF-β activity in endothelial cells [51]. Comparing with the Model group, HE sections in the QFXY group showed less pulmonary consolidation, which means QFXY help alleviate lung tissue remodelling.

Asthma is featured by reversible airway obstruction. The lack of full reversibility in some asthmatic patients may be due to chronic airway remodelling [52]. It appears that inflammation and remodelling are interdependent processes that clearly influence the clinical long term evolution of asthma [53]. The ECM can act as a reservoir for an increasing number of growth factors. These growth factors can be rapidly released from the ECM to allow extracellular signaling regulated by the growth factors to proceed without the need for new protein synthesis [54].

In QFXY-asthma target network, Hsp90α, Mapk3, VIM were hub proteins suggesting that they may be some targets of QFXY pills. The complicated interaction network suggested that QFXY pills affected a complex system regulating inflammation and immune reactions. Seen from the above complex network, QFXY interacts with asthma related genes in both direct and indirect way, affecting several signal pathways. In the previous study, 55 ingredients have been identified, including 27 absorbable constituents in QFXY, among which there are 19 ingredients affect inflammatory pathways, typically they are sulfur containing alkynes, such as arctic acid; lignans, such as arctigenin; phenolic acids, such as sinapic acid; steroids, such as cholic acid [13]. In the following study, other effects of these ingredients, such as alleviating airway hyperresponsiveness (AHR) and airway tissue remodelling will be further explored.

## Conclusions

A primarily combined genomic and proteomic screen of QFXY targets displayed a series of candidate genes and proteins, which indicated that the effect of QFXY relied on combined mechanism, anti-inflammation and anti-remodelling, as well as influence signal transduction in vivo.

## Competing interests

The authors declare that they have no competing interests.

## Authors' contributions

ZZ, GB and HW contributed to the conception and design of the study. ZZ completed the experiment. YM assisted the bioinformatics, PP finished 2D-MS, BC assisted the animal models. ZZ and HW drafted the manuscript. All authors contributed to further writing of the manuscript. All authors read and approved of the final manuscript.

## Pre-publication history

The pre-publication history for this paper can be accessed here:

http://www.biomedcentral.com/1472-6882/13/206/prepub

## Supplementary Material

Additional file 1 List of diff genes of microarrays.xls Click here for file

Additional file 2 Blasting list of diff genes.xls Click here for file

Additional file 3 GO analysis of diff genes.xls Click here for file

Additional file 4 KEGG pathways analysis of diff genes.xls Click here for file

Additional file 5 List of commonly shared pathway (sourced from KEGG, GenMAPP, and BioCarta) by diff genes and diff proteins.doc Click here for file
