# Supplementary material for: Qingfei Xiaoyan Wan alleviates asthma through multi-target network regulation
Source: BMC Complement Altern Med. 2013 Aug 6;13:206. doi: 10.1186/1472-6882-13-206 (PMC3765495; doi:10.1186/1472-6882-13-206)

| **Pathway** | **p-Value** | **q-Value** | **Gene** | **Protein** |
| --- | --- | --- | --- | --- |
| Contributed--cellular_process--Hs_TNF-alpha-NF-kB_NetPath_9 | 0.097238264 | 9.90E-04 | RPL6 | HSP90AA1 |
| Prostate cancer pathway | 0.046949559 | 6.50E-04 | MAPK3 | HSP90AA1 |
| GO_Samples--Cellular component--actin cytoskeleton | 0.106103671 | 0.001066369 | TNNT2 | CAPG |
| GO_Samples--Cellular component--endomembrane system | 0.098721671 | 9.97E-04 | SYNE1 | LMNB1 |
| GO_Samples--Cellular component--intermediate filament | 0.049032546 | 6.65E-04 | VIM | LMNB1 |
| GO_Samples--Molecular function--actin binding | 0.107083541 | 0.001070835 | SYNE1 | CAPG |
| GO_Samples--Molecular function--unfolded protein binding | 0.003161599 | 2.02E-04 | CRYAA;CRYAB | HSP90AA1 |


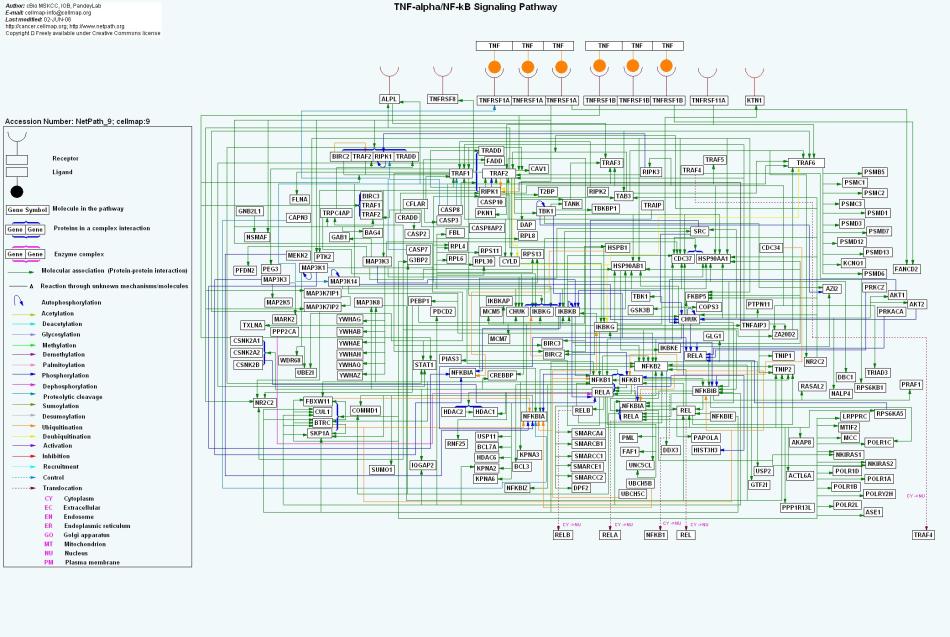


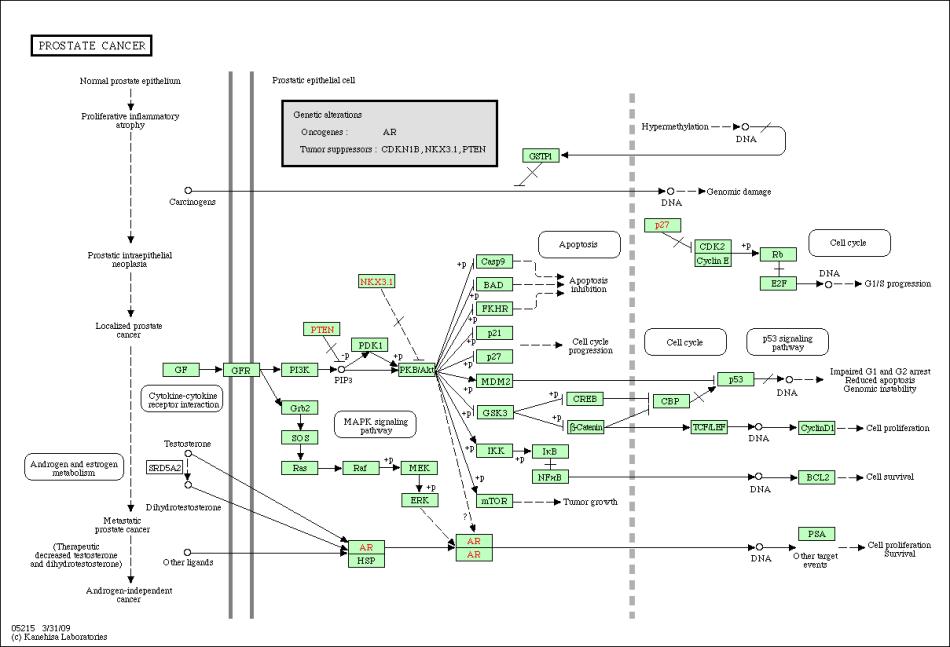


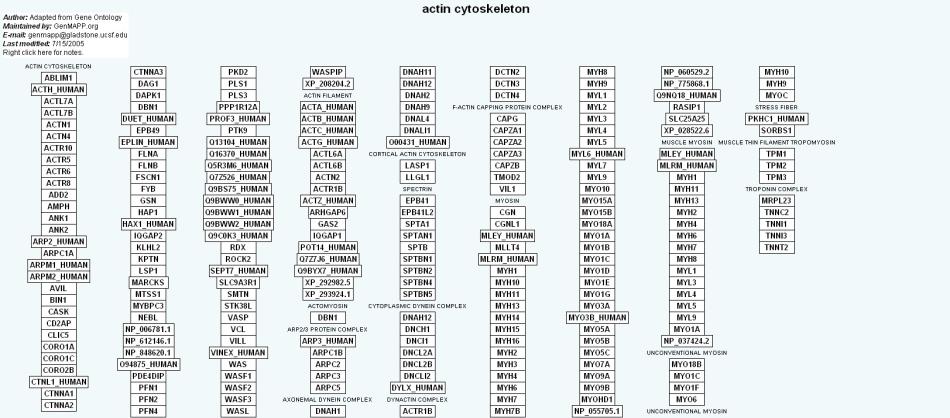


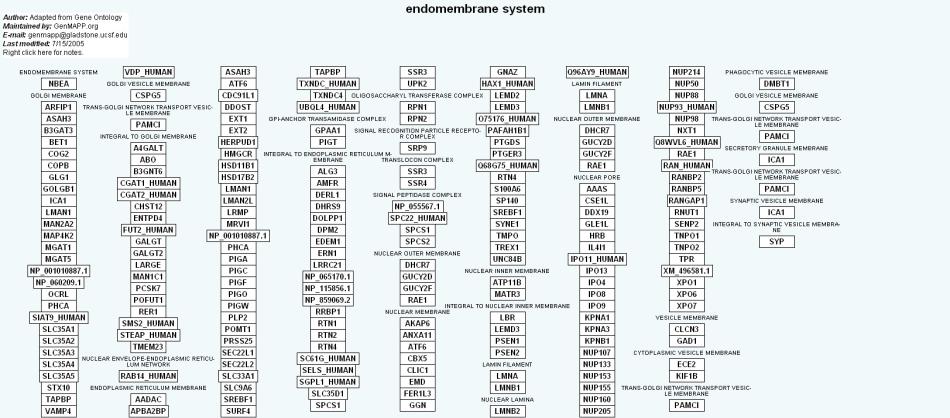


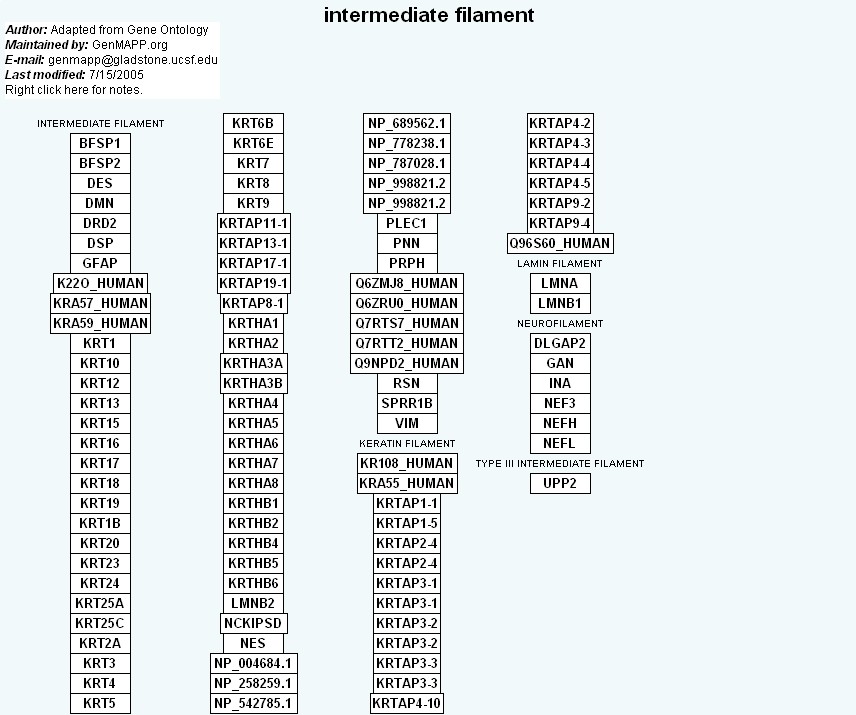


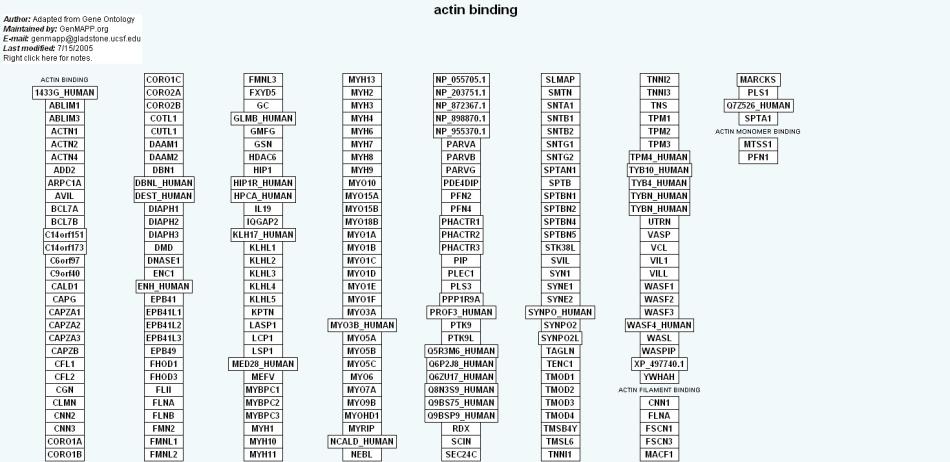


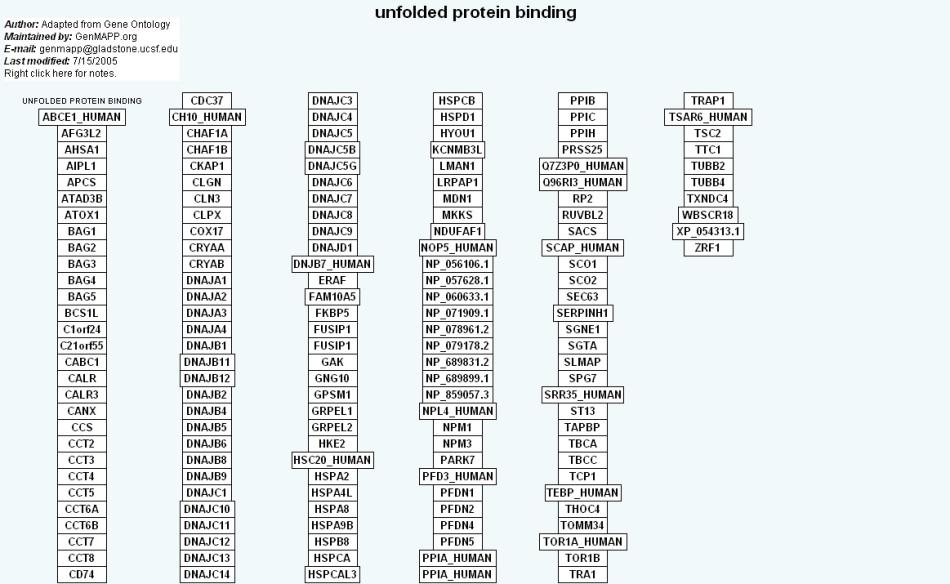

Supplement: Additional file 5 — List of commonly shared pathway (sourced from KEGG, GenMAPP, and BioCarta) by diff genes and diff proteins.doc [file 1472-6882-13-206-S5.doc]
